# Impaired Responses to In Vitro Lipopolysaccharide-Induced Stimulation After Long-Term, Rotating Shift Work

**DOI:** 10.3390/ijerph22050791

**Published:** 2025-05-17

**Authors:** Denise M. Jackson, Oscar Castanon-Cervantes

**Affiliations:** Department of Neurobiology and Neuroscience Institute, Morehouse School of Medicine, 720 Westview DR SW, Atlanta, GA 30310, USA

**Keywords:** long-term shift work, rotating schedules, chronic systemic inflammation, lipopolysaccharide, LPS response

## Abstract

Shift work is a common labor practice affecting nearly 30% of the U.S. workforce. Long-term, rotating-shift work is particularly harmful to health. Persistent sleep deprivation in shift workers, among other factors, facilitates the development of a state of subclinical but chronic systemic inflammation with a high incidence and prevalence of infections and inflammation-related pathologies, suggesting an underlying disruption of immune responses. However, despite this state of chronic immune activation, cell-mediated inflammatory responses in rotating-shift workers are poorly understood. Here, we used lipopolysaccharide (LPS) to stimulate peripheral blood mononuclear cells (PBMCs) isolated from rotating-shift workers and healthy day-shift workers and investigate their immune responses. The results showed that PBMCs from rotating-shift workers had a dampened inflammatory response. Specifically, the secretion of LPS-induced TNF-α in culture supernatants was significantly reduced compared to the response found in PBMCs from day-shift workers. However, anti-inflammatory responses, reflected by the secretion of LPS-induced IL-10, were indistinguishable between PBMCs from day-shift and rotating-shift workers. In addition, the correlation between the plasma concentration of lipopolysaccharide-binding protein (LBP, a marker of systemic inflammation) and LPS-induced responses was disrupted only in rotating-shift workers, suggesting that in this group, an impaired mechanism that weakens the relationship between pro- and anti-inflammatory signaling may underlie the hypo-responsiveness of PBMCs. Our results suggest that persistent subclinical systemic inflammation in rotating-shift workers disrupts cell-mediated immunity, increasing the risk of infection and other inflammation-related pathologies in this population.

## 1. Introduction

Rotating-shift work (broadly defined as any schedule where workers cycle between different daytime and night shifts periodically) is prevalent in an industrialized society that exposes workers to a disruptive environment of disturbed sleep and social interactions, as well as misaligned circadian rhythms and meal schedules [1,2,3]. According to the National Health Interview Survey and the Occupational Health Supplement, 27% of all workers in the United States work alternative (non-standard) working shifts, while 2.4% of the working population works rotational shifts [4]. The increased homeokinetic changes associated with chronic sleep deprivation and chronic circadian misalignment favor the conditions for significant health challenges to shift workers.

The effects of shift work on health derive from a complex mix of variables, including at least shift frequency (number of nights per week) and shift direction (clockwise vs. counterclockwise rotation). These variables disrupt two fundamental physiological functions (sleep and circadian rhythms). Extended disruption, even for those workers in permanent night shifts who rotate back to diurnal sleep habits on days off, but particularly for rotational shift workers, has been associated with chronic/partial sleep deprivation, increased stress, anxiety, and irregular menstrual cycles, among other conditions [5,6,7,8,9,10]. In addition, shift workers often struggle with proper nutrition and exercise, further exacerbating inflammatory responses and reducing resilience against pathogens.

Thus, research has shown that shift workers are more likely to be overweight or obese [10,11,12] and to develop subclinical (low-grade) systemic inflammation [13,14,15,16,17]. Despite a state of chronic activation of the immune system, shift workers are more likely to acquire infections [18,19,20] and develop diabetes, cardiovascular disease, stroke, several cancers, and autoimmune disorders such as lupus erythematosus, rheumatoid arthritis, inflammatory bowel disease, and multiple sclerosis [21,22,23,24,25,26,27,28].

The increased incidence and prevalence of these pathologies among shift workers suggest that disrupted inflammatory processes with aberrant cytokine production and impaired innate and adaptive immune responses may be present. However, finding functional outcomes as objective indicators of shift-work-related immune disruption has proven elusive. This is perhaps due to the diversity of cohorts, experimental approaches, and shift-work-relevant factors in interaction, making the isolation of single variables a truly challenging endeavor.

Nonetheless, studies have reported the effect of shift-work exposure on specific variables associated with immune disruptionsuch as increased monocyte, lymphocyte, and neutrophil counts, significant changes in cytokine release, and impaired inflammatory responses to LPS activation [29,30,31,32,33,34]. LPS, a potent bacterial product recognized by a typical pattern recognition receptor (TLR4), triggers innate immune responses and represents an objective tool to assess the functionality of the immune system in vivo, ex vivo, and in vitro [35,36,37,38].

Slightly elevated levels of LPS prevail in humans under unresolved or chronic inflammation [39,40,41]. Similarly, in shift workers, chronic, low-grade systemic inflammation is associated with slightly elevated levels of inflammatory markers, including LBP [34]. This acute-phase protein binds to LPS and serves as a bridge between bacteria and the immune system [42]. LBP is considered a proxy for LPS, and elevated LPS levels have been associated with the pathogenesis of several illnesses [43,44,45,46,47]. Thus, in long-term shift workers on rotating schedules, the development of subclinical but chronic and significantly elevated levels of inflammatory markers, including LBP, could lead to low-grade systemic endotoxemia.

The consequences that real-life rotating-shift work and chronic unresolved inflammation have on functional outcomes of immune effector cells remain poorly understood. Thus, this study assessed LPS-elicited responses in cultured peripheral blood mononuclear cells (PBMCs) isolated from subjects with a history of chronic exposure to rotating-shift schedules and healthy day-shift workers. Since we know that chronic exposure to low-grade systemic inflammation increases disease risk and leads to chronic immune activation [48], the potential changes in immune reactivity uncovered by this study may help better understand if the effects of long-lasting exposure to shift-work schedules on immune responses can be assessed before pathology ensues.

## 2. Materials and Methods

### 2.1. Study Groups

Nurses (14 rotating-shift workers and 12 day-shift workers) from Atlanta-area hospitals were screened and recruited by a self-reported intake survey about general health and history of shift-work exposure. All subjects were non-smokers and non-alcohol drinkers, free of any prescription drugs, with no clinical evidence of acute infection, sleep-related disorder, underlying immune-related pathology, or chronic illness.

The parameters for eligibility were based on published research [49,50,51]. Briefly, night work included at least six hours of work between 7 pm and 7 am, with no less than five night shifts per month (regardless of shift direction). Day-shift workers (control group) worked standard morning shifts (from 7 am to 7 pm), had no exposure to rotating-shift work in the three years before the study, no more than six months of exposure to shift-work schedules in a lifetime, and no travel history across more than one time zone during the three months before the study. All the subjects in this study provided written informed consent.

Prior to blood collection and to minimize the effects of time-of-day variations due to inconsistent diurnal rhythms and sleep patterns, subjects from both groups were required to maintain a regular sleep–wake schedule with bedtime between 11 pm and 6 am for seven consecutive days (three off days plus four morning-shift days). This schedule was verified by activity monitoring with a wrist-worn actigraphy tracker (Actigraph LLC, Pensacola, FL, USA). Then, blood collection for both groups occurred on the last day of these 7 days (during the last day of a 4-day morning shift) and at a fixed time window (8 am to 10 am).

### 2.2. Biospecimen Collection and LPS Stimulation

A total of 40 mL of venous blood was collected into EDTA-coated Vacutainer^®^ tubes (BD Biosciences, Bedford, MA, USA) from each subject. Two equal-volume aliquots of each sample were prepared. The first aliquot was used to determine the acute-phase reaction biomarkers. Whole blood was centrifuged at 1500× *g* for 10 min at room temperature; separated plasma was frozen at −80 °C until processed for analysis. The second aliquot was used for LPS stimulation. PBMCs were separated by density gradient centrifugation of whole blood at 400× *g* for 30 min at room temperature. PBMCs collected from the interphase were washed with PBS, and the red blood cells were lysed with BD PharmLyse (BD Biosciences) for 5 min at room temperature. Then, the cells were washed and resuspended for cryopreservation in RPMI-1640 medium, with 10% heat-inactivated FBS, 2 mM L-glutamine, 25 mM HEPES, 50 U/mL of penicillin/streptomycin, and 10% DMSO. PBMCs were frozen overnight at −80 °C using a freezing container, CoolCell^®^ (Corning, Somerville, MA, USA). The next day, cells were transferred into liquid nitrogen for storage. For culture and LPS stimulation, cryopreserved cells were thawed at 37 °C in culture medium (RPMI-1640 medium supplemented with 10% heat-inactivated FBS, 2 mM L-glutamine, 25 mM HEPES, and 50 U/mL of penicillin/streptomycin). After thawing, the cells were counted. The viability of thawed PBMCs, assessed by trypan blue exclusion, was 93.8%. The cells were immediately seeded at a final concentration of 1 × 10^6^ cells/mL in 24-well plates at 37 °C, 5% CO_2_, and a humidified atmosphere. Based on a dose–response curve to LPS stimulation (see Supplemental Materials), the final LPS (Sigma-Aldrich) concentration selected for stimulation of PBMCs was 1 µg/mL. Supernatant (200 µL) from the LPS-stimulated cells and non-stimulated controls was collected once at 24 h for later analysis of cytokine release.

### 2.3. Quantitative Assays

The concentration of inflammatory biomarkers in plasma (C-reactive protein (CRP), LBP, soluble-CD14 (sCD14), TNF-α, IL-10) and LPS-induced cytokine release from cultured PBMCs (TNF-α, IL-10) was determined by ELISA using commercially available kits following the manufacturer’s protocols. The sensitivity of the ELISA assay was 0.022 ng/mL, 0.47 ng/mL, 125 pg/mL, 6.23 pg/mL, and 3.9 pg/mL for CRP, LBP, sCD14, TNF-α, and IL-10, respectively. With the exception of LBP (bio-techne), the ELISA assays were purchased from R&D Systems.

### 2.4. Data Analysis

Based on the TNF-α-LPS-induced responses from cultured cells of healthy donors and shift workers, we estimated the required sample size using Cohen’s d and a *t*-test power analysis with the following parameters: a significance level (α) of 0.05, a desired power (1-β) of 0.99, and an estimated effect size of 1.71. The calculation indicated that a sample size of 15 per group was sufficient to detect a statistically significant difference. A sample size of 12 was powered at 98%. All calculations and statistical tests were performed with SAS 9.4. Normality distribution was assessed by the Shapiro–Wilk test. For an analysis of categorical data, we used Fisher’s exact test, and for an analysis of continuous data, including the assessment of changes in the LPS-induced cytokine release between day-shift and rotating-shift groups, a two-tailed *t*-test for independent samples or the Mann–Whitney test was used. The Wilcoxon signed-rank test determined the significant difference between treatments (stimulated vs. non-stimulated). Finally, we assessed the strength and direction of the relationship between baseline systemic inflammation and in vitro cytokine release with a Pearson correlational analysis. Data are expressed as mean ± standard error. Across all analyses, a statistically significant difference was accepted as *p* < 0.05.

## 3. Results

### 3.1. Group Characteristics and Assessment of Immune Activation

Going into the study, the recruited subjects had similar BMIs, ages, gender compositions, and health histories. Thus, we worked carefully to account for other shift-work-relevant variables between the groups that we could control and that might affect inflammation. All the shift workers in the study reported changes in the direction (clockwise vs. counterclockwise) and frequency (twice, three, or four times per week) of the rotations throughout their tenure (i.e., to make healthcare more efficient and improve employee satisfaction, different scheduling systems have been tested by hospitals over time). These changes in specific aspects of the schedule of each participant over two decades of work could not be documented. However, despite this potential variation in individual experiences, each participant consistently worked rotational shifts for at least 15 years (mean exposure = 20.86; range = 15–26 years), and therefore, was chronically exposed to the effects of sleep deprivation, circadian misalignment, and social stress on innate and adaptive immune responses. Other characteristics of the study groups are indicated in Table 1.

In order to assess the degree of systemic inflammation in the study groups, we first determined the concentration of specific acute-phase response biomarkers that provide an objective indication of the state of immune activation. The results show that the assessed biomarkers were significantly higher in rotating-shift workers (all *p* < 0.01), indicating subclinical systemic inflammation. We found that, compared to day-shift workers, rotating-shift workers had a CRP concentration that was 66% higher. Similarly, rotating-shift work increased the concentration of LBP, which was 28% higher, and sCD14, which was 79% higher than the concentration found in day-shift workers. Baseline TNF-α and IL-10 concentrations in plasma were also significantly increased in rotating-shift workers compared to day-shift workers (see Table 2).

### 3.2. LPS-Induced Cytokine Secretion

**Figure 1:**
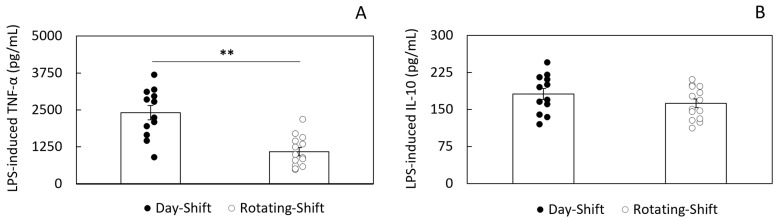
Cytokine secretion in PBMCs isolated from day-shift and rotating-shift workers after stimulation. (**A**) LPS-induced TNF-α; (**B**) LPS-induced IL-10. Individual data points are shown. The columns show the average LPS response for each group ± standard error (*p* < 0.01 **).

The significant increases found in acute-phase reaction and immune activation biomarkers suggested a state of low-grade systemic inflammation in rotating-shift workers with the potential to disrupt cell-mediated immunity. To test this hypothesis, we isolated PBMCs from both day-shift and rotating-shift workers and evaluated the responsiveness of these cells to LPS stimulation. Specifically, we assessed LPS-induced secretion of TNF-α (pro-inflammatory) and IL-10 (anti-inflammatory). PBMCs from both groups showed characteristic increases in TNF-α and IL-10 release in response to LPS. However, we found a significant difference in the response to LPS between day-shift workers and rotating-shift workers that was cytokine-specific (see Figure 1). When compared to day-shift workers, the concentration of TNF-α was significantly lower in rotating-shift workers (1087.1 ± 136.76 vs. 2407.02 ± 237.88 pg/mL, *p* < 0.01). In contrast, the concentration of LPS-induced IL-10 was indistinguishable between rotating-shift workers and day-shift workers (181.49 ± 11.22 vs. 162.49 ± 8.64 pg/mL, *p* = 0.19). Non-stimulated PBMCs showed minimal and no significant difference in cytokine release between the groups (see Supplemental Materials).

### 3.3. Correlation Between Immune Activation and LPS-Induced Responsiveness

Next, we sought to investigate if the disrupted responses to LPS stimulation found in PBMCs from rotating-shift workers were related to the in vivo state of immune activation. Because LBP is mainly produced in the liver, it is not directly involved in the in vitro response to LPS, and it is a well-established marker of in vivo cell-mediated immune activation, we conducted a correlational analysis to assess the relationship between the concentration of plasma LBP and the magnitude of the LPS response. In PBMCs from day-shift workers, we found a high, positive correlation between the plasma LBP concentration and the LPS-induced levels of TNF-α and IL-10. On the contrary, in PBMCs from rotating-shift workers, the correlation between the LBP concentration and the LPS-induced TNF-α was negligible, while a positive correlation between LBP and LPS-induced IL-10 remained high. No significant correlations were found between plasma sCD14 and LPS responses. Table 3 summarizes the results of the Pearson correlational analysis.

## 4. Discussion

In this study, we aimed to investigate whether disrupted immune reactivity may be a factor associated with an increased risk of infections and other inflammation-related pathologies in rotating-shift workers despite a chronic state of immune activation. The increases in inflammatory markers reported in this study are not high enough to be considered pathological. In other words, these are all subclinical levels, or what is typically called low-grade systemic inflammation. The chronic nature of this unresolved inflammatory state represents a serious challenge to health, ultimately leading to cellular reprogramming of immune responses and pathology. We present here evidence of these changes in inflammatory responses with a cell-mediated immunity assay from cells of exposed individuals who are still asymptomatic. We show that the LPS-induced responses of PBMCs isolated from rotating-shift workers are clearly disrupted. Specifically, cultured cells from rotating-shift workers failed to mount a robust LPS-induced TNF-α response. By contrast, their anti-inflammatory response to LPS, as indicated by the assessed secretion of IL-10 into the culture’s supernatant, was indistinguishable from the response found in PBMCs from healthy day-shift workers. Furthermore, we found that the high and significant correlation between plasma LBP (an objective marker of systemic inflammation and immune activation) and LPS-induced cytokine secretion from PBMCs was significantly disrupted only in rotating-shift workers. This effect was cytokine-specific and only affected LPS-induced TNF-α, indicating a disturbance in the forces that modulate the delicate balance between pro- and anti-inflammatory signaling required to mount an efficient immune defense. Both long-term shift-work exposure and rotating schedules have been recognized as two of the most detrimental variables linked to pathology [2,3,4,5,6,7,8,9,10].

As indicated before, the groups in this study had similar characteristics (see Table 1) except for shift-work history. It is in this context that PBMCs from rotating-shift workers secreted significantly less LPS-induced TNF-α despite increasing plasma levels of this and other inflammatory markers. These observations suggest that in groups with similar BMIs (both groups were slightly overweight), ages, gender compositions, and health histories, the length of exposure to rotating schedules is strongly associated with the disruption of immune reactivity. We cannot attribute this effect to a single factor, especially since it is well-known that other shift-work-relevant variables (i.e., chronic sleep loss, irregular diet, disrupted social interactions, increased stress, and circadian misalignment) also affect the immune response. However, the evidence supporting the significant effect of length of exposure (from acute to chronic) is well documented [52,53,54,55,56,57].

Thus, if the disruption of inflammatory responses in association with chronic systemic inflammation is a common factor among many pathologies, such as diabetes [39,58], obesity [58,59], sepsis [60], and others [22,48,61], it is reasonable to hypothesize that in rotating-shift workers, given enough time, similar changes in functional outcomes of cell-mediated immunity may eventually develop and explain the increased risk of infection among this population.

Binding of LPS with LBP is the first step in activating the CD14:TLR4 pathogen recognition pathway [42,62], and even subclinical amounts of LPS in circulation are considered evidence of systemic endotoxemia [63,64]. We assessed LBP, a proxy for LPS [46,47], which is more stable in the bloodstream and more straightforward to measure than LPS [65]. Notably, we found that rotating-shift workers had significantly elevated levels of LBP and sCD14 in plasma. The presence of these biomarkers of immune activation cannot be understated and prompted us to investigate cell-mediated inflammatory responses.

The significant increase in plasma LBP and sCD14 from rotating-shift workers is clearly strong evidence of monocyte activation [66,67,68]. However, we did not aim to identify a unique cell phenotype responsible for impaired inflammatory responses, as innate immune reactivity is not a function of only monocytes, B, or T cells. Since the activation state of monocytes can be changed by the isolation procedure [69], and their responses to LPS are mediated by their interaction with other cells in circulation, we used a PBMC preparation that included monocytes, lymphocytes (LPS-responding B and T cells), and dendritic cells that afforded the opportunity to investigate LPS-induced activation in an environment that preserved many of the interactions prevailing in vivo and minimized the effects of the isolation protocol on the activation state of the cells [70,71,72].

Studies have found that systemic inflammation may trigger the reprogramming of innate and even adaptive immune responses [61,73]. One of the mechanisms by which reprogramming of immune responses occurs is known as endotoxin tolerance, which may help explain the seemingly paradoxical observation of impaired responses to LPS stimulation by an overactive immune system [74,75,76]. Our results are consistent with the hypothesis of endotoxin tolerance. We found that the increase in LBP and sCD14 in rotating-shift workers, as evidence of chronic immune activation, is strongly associated with the disruption of LPS-induced responses. Similar mechanisms resembling endotoxin tolerance have been proposed for the relationship between chronic inflammation and impaired immune responses [39,43,73,75,77,78].

Endotoxin tolerance has been thought of as a protective mechanism of the immune system against excessive inflammation [78]. LBP and LPS play a significant role in endotoxin tolerance. Exposure to LPS, in either high or low doses, triggers cellular reprogramming and changes the response of immune cells to a future LPS encounter [45]. These effects are complex and are not limited to the effect of LPS on monocyte activation. Both LBP and sCD14 play important roles in the signal transduction pathways modulated by their abundance and the environment in which they function [79].

One of the effects of endotoxin exposure in vivo is the downregulation of membrane-associated CD14 (mCD14) in peripheral blood monocytes. Downregulation of mCD14 through shedding or secretion results in an increased sCD14 [80]. Although we did not assess the downregulation of mCD14 directly in our preparation, we saw plasma levels of sCD14 increase. In human serum or plasma from healthy subjects, sCD14 is found at a concentration of 2–3 μg/mL, but increased levels typically paired with reduced expression of mCD14 in monocytes have been associated with several inflammatory and infectious pathologies [81,82,83,84,85]. In addition, sCD14 can inhibit cell-mediated responses by facilitating LPS transfer from mCD14 to plasma lipoproteins [86]. This ability of sCD14 to act as a scavenger and elicit immune responses beyond its interaction with LBP (both in vivo and in vitro) has been shown in cells lacking mCD14 (other than monocytes) that can still respond to LPS [87].

Despite the reduced secretion of LPS-induced TNF-α by PBMCs in rotating-shift workers, their secretion of IL-10 was comparable to that found in day-shift workers (see Figure 1). Although we did not seek to determine the specific source of LPS-induced IL-10 secretion in our preparation, we know that monocytes and B cells are primary sources of this anti-inflammatory cytokine [88]. Notably, it has been previously reported that higher levels of sCD14 can enhance B cell activation [89]. By binding directly to most peripheral blood B cells, including regulatory B cells, sCD14 may also play a pivotal role in regulating anti-inflammatory signaling. Thus, in an environment where LBP and sCD14 are increased, and mCD14 is downregulated, B cells are more likely to secrete IL-10. The abundance of sCD14 and LBP can shift the immune response towards a more anti-inflammatory state, promoting the secretion of regulatory cytokines like IL-10.

In this context, endotoxin tolerance could explain how PBMCs from shift workers would respond to a weaker signal to activate inflammatory responses (prompted by excess in vivo exposure to LBP:LPS), and instead, show a relative increase or no change in anti-inflammatory signaling. In pathologies with a common inflammatory component, and as diverse as obesity, diabetes, sepsis, cystic fibrosis, and COVID-19, several studies have reported a diminished capacity to release pro-inflammatory cytokines at the same time as an enhanced or unchanged anti-inflammatory response [76,90,91,92,93].

Although this refractory state characteristic of endotoxin tolerance is a protective mechanism, it can also become a challenge if the forces that control the balance between pro- and anti-inflammatory signaling are disturbed by too much anti-inflammatory signaling. Under chronic sleep deprivation, as is the case for long-term exposure to rotating-shift work, a perpetual state of unresolved systemic inflammation with excessive anti-inflammatory signaling may promote pathogen persistence and increased susceptibility to infection [94].

In the end, the effects of shift work on health are pervasive and range from increased absenteeism [95] to a higher susceptibility to infections and reduced vaccine efficacy. Long-term, the impact on immunity is also significant. Disrupted sleep patterns, circadian rhythms, and chronic systemic inflammation contribute to chronic diseases like cardiovascular disease, diabetes, and even cancer [13,14,15,16,17,18,19,20,21,22,23,24]. Aside from further research on biomarkers of shift-work-related disease, the implementation of effective strategies for managing inflammation in shift workers should be a priority. These strategies could include maintaining consistent sleep schedules that minimize circadian disruption, prescribing anti-inflammatory medication, and engaging in regular physical activity.

## 5. Conclusions

Our results provide a path to uncover the biological significance of persistent subclinical systemic inflammation in rotating-shift workers as a disruption of a functional outcome in PBMCs. Such changes in cell-mediated immunity may contribute to the increased risk of infection in shift workers. Subtle but consistent and significant changes in immune reactivity may also serve as early warning signals for those workers exposed to the detrimental effects of rotating shifts over decades. However, since susceptibility to low-grade systemic inflammation, sleep deprivation, circadian misalignment, and social stress may vary among individuals, it is important to consider the implementation of screening practices that could identify impaired inflammatory responses early on. Low-cost, minimally invasive health-surveillance interventions are key to allowing shift workers to continue their activity [96]. These functional outcomes can be assessed before pathology diagnosis, thus increasing the opportunity to identify at-risk individuals and changing the nature of shift-work-related medicine from reactive to proactive and preventive.

### Limitations

This study has important limitations that may impact the significance of our findings. These include a small sample size and overrepresentation of female workers and the healthcare sector. These limitations restrict the generalizability of the findings to a more diverse population. Because we did not gather information on chronotypes, our study may overlook important individual differences in sleep–wake patterns and biological rhythms that can influence inflammatory outcomes. The irregularity of rotational schedules (i.e., constant changes in frequency and direction of rotation) over the past two decades in our group of shift workers made a more granular analysis of specific aspects of the schedules not feasible. Therefore, the ability to compare our results to lab-based studies that simulate short-term shift-work schedules or field studies that investigate the independent effects of multiple work schedules on immune-related outcomes is limited.

Although significant and consistent with the hypothesis of endotoxin tolerance, the hypo-responsiveness of PBMCs from rotating-shift workers we report here is only evidence of a potential relationship between low-grade systemic inflammation, length of shift-work exposure, and impaired immune reactivity. However, the direct association or causal effect remains to be elucidated and would require a larger sample size, and assessing changes in the expression of the TLR4 and mCD14 receptors. In addition, an accurate assessment of immune status, where the cellular proportion of monocytes, B, and T cells is considered, could significantly influence the results. Our current approach is not precise enough to ensure that the observed differences in cytokine release are attributable to true immune system variations rather than differences in cell proportion. Future studies will address these limitations.

## Figures and Tables

**Table 1:** Study groups.

	Day Shift (*n* = 12)	Rotating Shift (*n* = 14)
BMI (Kg/m^2^)	25.97 ± 0.42	26.1 ± 0.26
Age (years)	47.75 ± 1.95	45.64 ± 1.71
Gender (% female)	83.3	78.6
Shift-work exposure (years) **	0	20.86 ± 1.11

Except where indicated, values are expressed as means ± standard error. Statistically significant difference (*p* < 0.01 **) between the groups.

**Table 2:** Acute-phase reaction and immune activation biomarkers.

	Day Shift (*n* = 12)	Rotating Shift (*n* = 14)
CRP (mg/L) **	1.85 ± 0.16	3.08 ± 0.21
LBP (µg/mL) **	5.95 ± 0.2	7.6 ± 0.26
sCD14 (µg/mL) **	2.54 ± 0.34	4.55 ± 0.33
TNF-α (pg/mL) **	14.93 ± 0.68	19.64 ± 0.91
IL-10 (pg/mL) **	2.23 ± 0.18	5.71 ± 0.29

Values are expressed as means ± standard error. Statistically significant difference (*p* < 0.01 **) between the groups.

**Table 3:** Correlation between plasma LBP and cytokine release from PBMCs.

	LPS-Induced TNF-α	LPS-Induced IL-10
Plasma LBP	Day Shift: *r* (10) = 0.87, *p* =0.0002 ** Rotating Shift: *r* (12) = 0.18, *p* = 0.54	Day Shift: *r* (10) = 0.78 *p* = 0.003 ** Rotating Shift: *r* (12) = 0.67, *p* = 0.009 **

Pearson correlational coefficient (*r*), degrees of freedom (in parenthesis), and *p*-values are indicated; *t*-test (two-tailed) was used to assess the significant difference of the correlation **.

## Data Availability

The original contributions presented in this study are included in the article/Supplementary Materials. Further inquiries can be directed to the corresponding author.

## Supplementary Materials

The following supporting information can be downloaded at: https://www.mdpi.com/article/10.3390/ijerph22050791/s1. Figure S1: Dose–response curve to 24 h LPS stimulation of cryopreserved PBMCs. Table S1: Cytokine secretion in collected supernatants 24 h after seeding (PBMC non-stimulated controls).

## Abbreviations

The following abbreviations are used in this manuscript:

LPS	Lipopolysaccharide
LBP	Lipopolysaccharide-binding protein
PBMCs	Peripheral blood mononuclear cells
sCD14	Soluble CD14
