# Supplementary material for: Impaired Responses to In Vitro Lipopolysaccharide-Induced Stimulation After Long-Term, Rotating Shift Work
Source: Int J Environ Res Public Health. 2025 May 17;22(5):791. doi: 10.3390/ijerph22050791 (PMC12110847; doi:10.3390/ijerph22050791)
Supplement: Supplementary file 1 [file ijerph-22-00791-s001.zip › ijerph-3555586-supplementary.pdf]

Supplemental Figure S1. Dose-response curve to 24h LPS-stimulation of cryopreserved PBMCs.

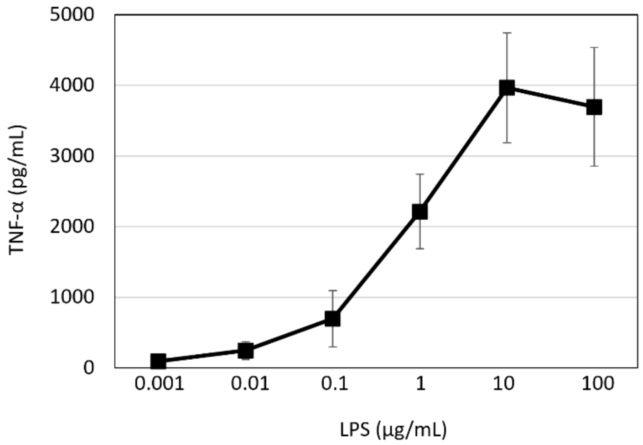

Supplemental Table S1. Cytokine secretion in collected supernatants 24 h after seeding (PBMC non-stimulated controls)

	Day-Shift	Rotating-Shift
TNF-α (pg/mL)	10.26 ± 0.87	10.95 ± 0.56
IL-10 (pg/mL)	5.06 ± 0.36	5.25 ± 0.33

Values are expressed as means ± standard error.
